# The Effects of Silica-Based Fillers on the Properties of Epoxy Molding Compounds

**DOI:** 10.3390/ma12111811

**Published:** 2019-06-04

**Authors:** Mitja Linec, Branka Mušič

**Affiliations:** 1 Kolektor Group d.o.o., Vojkova 10, 5280 Idrija, Slovenia; 2 Nanotesla Institut Ljubljana, Stegne 29, 1000 Ljubljana, Slovenia; branka.music@kolektor.com

**Keywords:** epoxy molding compound, fillers, silicon dioxide, flow spiral length, tensile strength

## Abstract

Global design and manufacturing of the materials with superb properties remain one of the greatest challenges on the market. The future progress is orientated towards researches into the material development for the production of composites of better mechanical properties to the existing materials. In the field of advanced composites, epoxy molding compounds (EMCs) have attained dominance among the common materials due to their excellent properties that can be altered by adding different fillers. One of the main fillers is often based on silicon dioxide (SiO_2_). The concept of this study was to evaluate the effects of the selected silica-based fillers on the thermal, rheological, and mechanical properties of EMCs. Various types of fillers with SiO_2_, including crystalline silica and fused silica, were experimentally studied to clarify the impact of filler on final product. Fillers with different shape (scanning electron microscope, SEM), along with different specific surface area (specific surface area analyzer, BET method) and different chemical structure, were tested to explore their modifications on the EMCs. The influence of the fillers on the compound materials was determined with the spiral flow length (spiral flow test, EMMI), glass transition temperature (differential scanning calorimetry, DSC), and the viscosity (Torque Rheometer) of the composites.

## 1. Introduction

Epoxy molding compounds (EMCs) are commonly used in various thermosets applications, due to their low viscosity, high flow, good dimensional stability, and high productivity. They are demanded for many years in a multitude of industrial products, from highly advanced automotive and aerospace parts, coatings and adhesives for construction, to electrical and electronic implementations. Many of them are largely applied in microelectronics to protect semi-conductor devices from environmental hazards, such as moisture, chemical agents, dust, and external impact [1]. Therefore, different requirements, such as good adhesion to the base materials, low viscosity, good dimensional stability, and high chemical resistance, need to be obtained.

The properties of EMCs have been enhanced with production procedure modification or composites formulations variation. This often includes the introduction of dispersed toughened phase, such as micro sized particles in the polymer matrix [2,3]. The composition of EMCs always contains micro sized fillers of large amount (more than 50%), which are successfully used in the stiffening and strengthening of the epoxy molding product. The addition of filler could also lead to an increase of Young’s modulus, and thus in the lower elongation of the composite material [4,5]. For this reason, fillers with silicon dioxide are generally involved in the preparation of epoxy composite material [6]. With the proper choice of such type of filler, its right amount, and the method to integrate it into the selected composites, we can potentially improve their chemical, thermal, and electrical resistance qualities, mechanical properties, and dimensional stability [7,8]. These low cost components usually do not affect, or are able even to lower, the price of EMCs, but they can have a considerably better outcome on the properties of the final product.

In one of the approaches, they tried to reinforce EMCs with nano-sized silica particles, but they gained very slight enhancement in the viscosity of epoxy molding composite [9]. Furthermore, the focus was not set for large-scale composite utilizations. The mechanical property improvements that can be achieved by modifying epoxy molding compounds with silica and silicate based fillers, like hardness, flowability, and elasticity of the final product, have been the subject of many researches [10,11,12,13,14,15,16,17,18,19].

The aim of this work is to examine the advantages of fillers with silicon dioxide for a product that is aimed for industrial application. The focus was set on the properties of EMCs that were prepared with a variety of commercial available fillers with different specific surface area and chemical structure. We tried to explore the effects of fillers, especially on the thermal (glass transition temperature, T_g_), mechanical (tensile strength), and rheological (flow spiral length, viscosity) characteristics of epoxy resins based final product.

## 2. Materials and Methods

### 2.1. Materials

We applied four commercial available samples of fillers containing silicon dioxide, supplied with different manufacturers, marked as F1, F2, F3, and F4 (Table 1), for our work. The epoxy resin used was an o-cresol novolac based epoxy resin (213 g/epoxy equivalent). Phenol novolac resin (105 g/hydroxyl equivalent) and combination of 2-methylimidazole/dicyandiamide were used as a hardener and an accelerator for the curing of the epoxy resin, respectively. Along with silica-based filler (22.0 wt. %) in the formulation of EMCs, others components that were used in this study were glass fibers, inorganic fillers, mold release agents, and pigment.

### 2.2. Filler Properties

**Figure 1:**
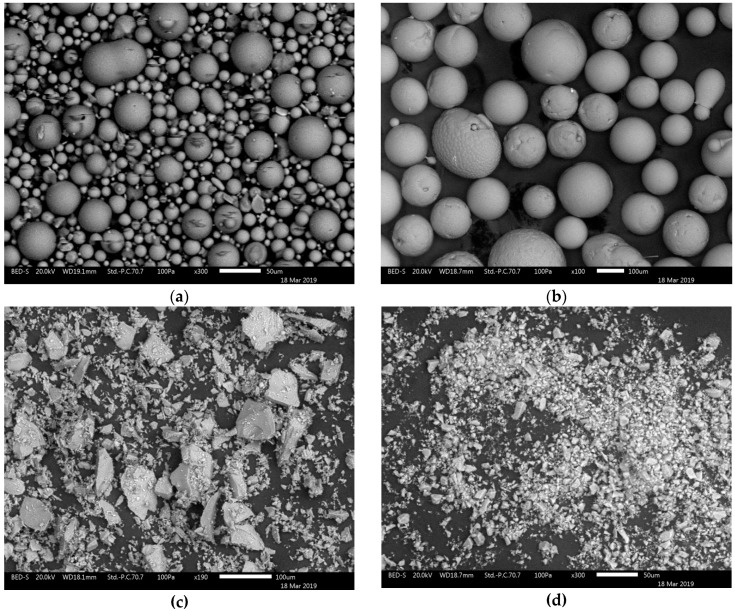
Scanning electron microscope (SEM) images of the tested types of fillers: (**a**) glass beads; (**b**) foundry sand; (**c**) crystalline silica; and, (**d**) fused silica.

Table 1 summarizes the bulk density, the specific surface area, and chemical content of SiO_2_ for all four fillers with silicon dioxide. The bulk density is significantly lower and specific surface area is noticeably larger than the rest of the fillers for fused silica filler. The properties of silicon dioxide consisted fillers investigated in this study are as follows:

(1) *Glass beads:* Glass beads are spherical shaped particles that are produced by the melting and molding of fine glass powder. With their round shape, we might obtain high loadings, and hence, stiffness, but in the absence of sharp, stress raising edges, we can also achieve better toughness retention. In this work, we used glass beads with particle size distribution between 0 µm and 50 µm, which is represented in the image from the scanning electron microscope (SEM, JSM-IT300LV, JEOL, Tokyo, Japan), as shown in Figure 1a.
(2) *Foundry Sand:* Foundry sand is a type of spherical ceramic casting sand with ball-shaped particles. It is made from bauxite by high-temperature melting in electric-arc furnace, spraying and cooling with the characteristics of high refractoriness and low thermal expansion. For this study, we applied particles between 50 µm and 200 µm, as shown in Figure 1b.
(3) *Crystalline Silica:* Crystalline silica is usually produced by chemical purification and mechanically crushing natural quartz. Its morphology has irregular shapes with sharp corners, as shown in Figure 1c with average size of 30 µm.
(4) *Fused Silica:* Fused silica is commonly made by melting crystalline silica at high temperatures of 1900–2500 °C, and then by crushing and pulverizing after re-solidification. Fused silica is amorphous powder, which is depicted in Figure 1d. The internal stress is released and the results in low density and in a smaller coefficient of thermal expansion than that of crystalline silica because of the melting and re-solidification processes.

### 2.3. Preparation of Epoxy Molding Compounds

**Figure 2:**
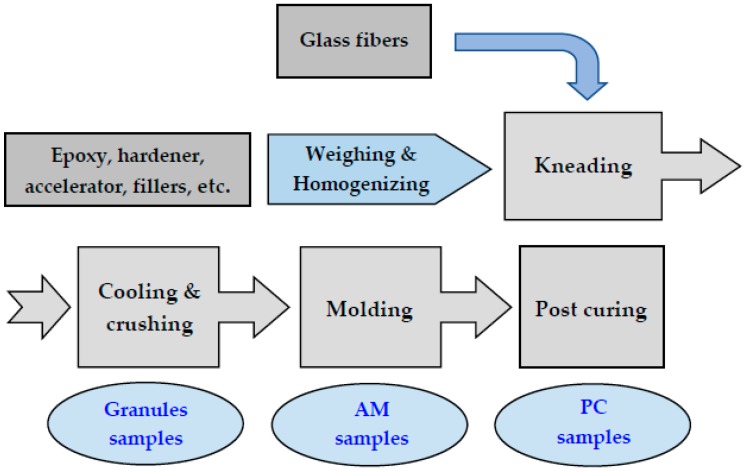
Stages for the preparation of epoxy molding compound.

The preparation procedure of EMCs incorporated series of operations, as shown in Figure 2. All of the components, except glass fibers, were thoroughly premixed in a high-speed mixer for 6 min. at room temperature to achieve a homogeneous mixture. The blend of homogeneous mixture and glass fibers were mixed and melt-kneaded in a continuous axial oscillating single-screw extruder (Buss PLK 46-11R, Buss AG, Pratteln, Switzerland) at 120 °C. After the kneading process, the material was calendered on the two roll calender machine (Kolektor iDrium, Ljubljana, Slovenia), air cooled, and crushed on the power cutting mill (Pulverisette 25, Fritsch, Idar-Oberstein, Germany) into the composite granulate of irregular shapes ranging from 3 mm to 4 mm in size.

The molding process of composite granulate with volatile organic compounds (VOCs) between 0.2% and 0.3% took place in a hot hydraulic press at (180 ± 5) °C with a pressure range from 180 bar to 200 bar. This resulted in a product of a hollow cylinder shape with the pre-determined dimensions. Further treatment of molded product was by a post curing thermal process at 210 °C for 5 h.

### 2.4. Testing Methods

Four different EMCs—identified as EMC1, EMC2, EMC3 and EMC4 (Table 2, Table 3, Table 4 and Table 5)—were prepared and tested, each with different filler containing silicon dioxide, marked as F1, F2, F3, and F4 (Table 2, Table 3, Table 4 and Table 5), respectively. The weight loading of each filler containing silicon dioxide was equal for all the prepared composite samples (22.0 wt. %) and the weight fraction of other used components remained consistent. The methods of determining the EMCs properties are as follows.

- *Differential Scanning Calorimetry (DSC):* The tested samples were heated in a thermal analyzer apparatus (DSC823^e^ Module STAR^e^ System, Mettler Toledo, Greifensee, Switzerland). We determined the heat flow profiles of EMC materials before the molding process, after the molding process, and after the post curing regime. Aluminum pans with pierced lids containing 5–10 mg of tested samples were analyzed with a temperature rate of 10 °C/min from 25 °C to 300 °C under air environment.
- *Rheology analysis:* The flow-curing behaviors of EMCs were measured using a torque rheometer (MB 30, Brabender, Duisburg, Germany). The tested samples were loaded into the heated mixer bowl at 150 °C, where were sheared by a triangle shaped mixing blades at 50 rpm for approximately 6 min. The torque of mixing blades that were connected to a drive unit was measured and recorded during the mixing process. The torque during the mixing process mirrors the resistance that the material opposes to the rotating blades, and it is measured as a function of time.
- *Flow spiral length:* Spiral flow is a standardized test (ASTM D3123) of epoxy molding compounds to check the flowability that is extensively used in the manufacturing. For this test, the flow spiral length of EMCs was examined in the testing machine Lauffer Pressen UVKO 25 (Horb am Neckar, Germany). Typically, the standard molding temperature and the transfer pressure are (150 ± 3) °C and 69 bar.
- *Specific surface area (BET method):* The specific surface area of fillers was measured while using the N_2_ adsorption-desorption BET method (TriStar 3000, Micromeritics, Norcross, GA, USA).
- *Density:* The densities of cured EMCs final test products were measured with a high precision analytical balance in water with a density kit attached (XP205 DeltaRange, Mettler Toledo, Greifensee, Switzerland) at room temperature.
- *Mechanical test:* The mechanical characterization of cured EMCs final test products was carried out using a static tensile machine (Z100, Zwick/Roell, Ulm, Germany). The tensile strength was analyzed on a hollow cylinder shaped samples, with the outer diameter 20.00 mm, inner diameter 12.00 mm, and height 15.00 mm.

## 3. Results and Discussion

### 3.1. Differential Scanning Calorimetry (DSC)

**Figure 3:**
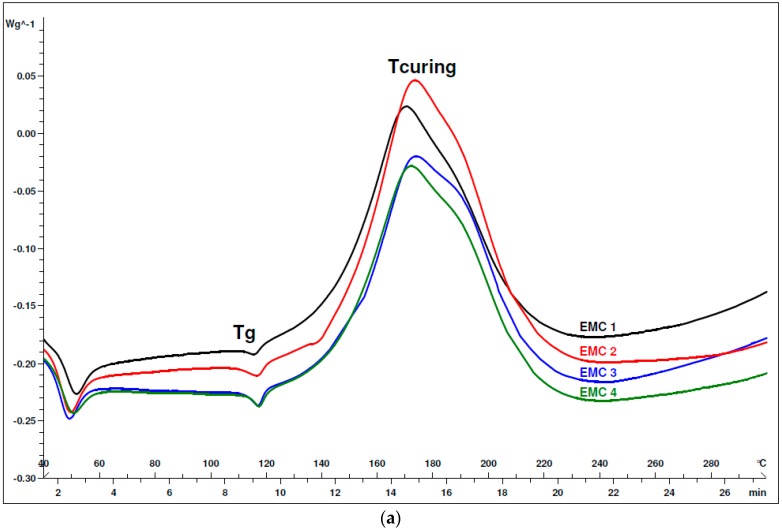

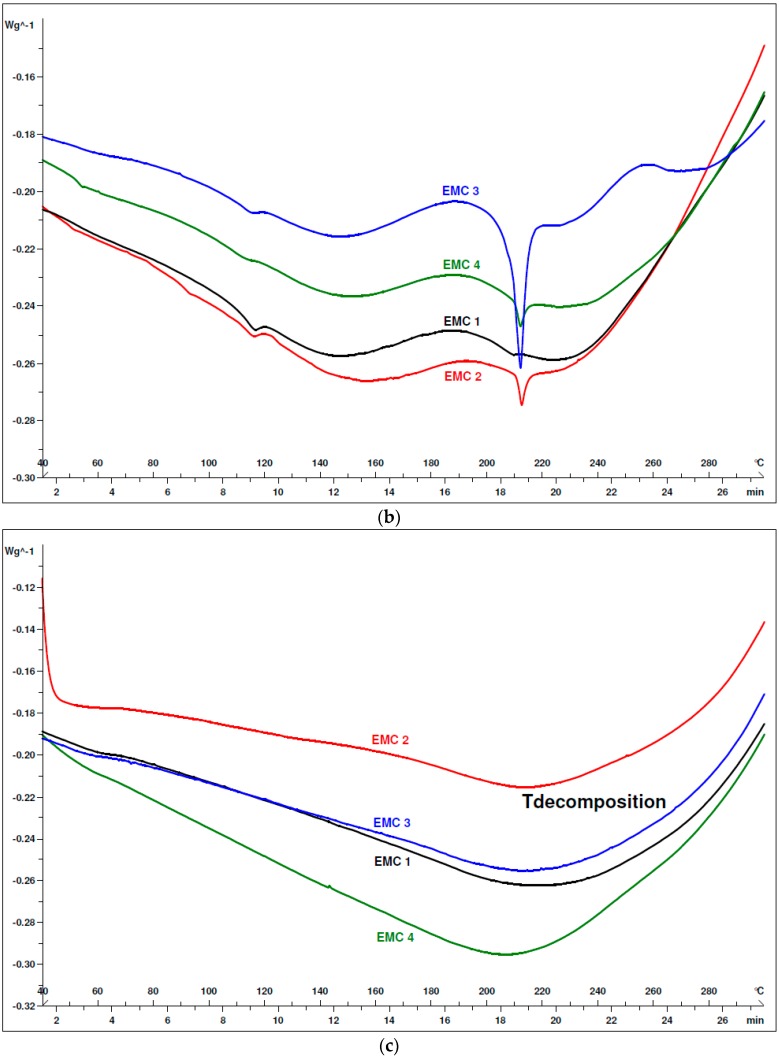
(**a**) Differential Scanning Calorimetry (DSC) thermogrames of prepared epoxy molding compounds (EMC) granulates before molding process; (**b**) DSC thermogrames of prepared EMC products after molding process; and, (**c**) DSC curves thermogrames of prepared EMC products after post curing process.

DSC measurements were performed in order to verify the curing profiles of EMC granulates that were prepared with different silica-based fillers. Figure 3a depicts the obtained thermogrames of each produced EMC granulate. For all of the prepared EMCs, the thermogrames are nearly the same and are independent from the used type of filler. The first endothermic peak at 50 °C is attributed to the softening point of epoxy resin, while the second endothermic peak at 113 °C represents the glass transition temperature (T_g_) of the EMC granulate. The hardening of epoxy resin is accomplished by phenol novolac resin through exothermic cross-linking of the functional groups of hardener and resin between 140 °C and 220 °C. In our case, the T_g_ of EMC granulates was difficult to measure, because it was in close proximity to the onset of the curing reaction at around 140 °C. The glass transition temperature and the peak temperature of curing process were approximately the same for all prepared EMC granulates, as presented in Table 2.

Figure 3b shows summary of the DSC results for the EMC molded products. The DSC thermogrames are still almost the same for all produced EMCs. The endothermic peak at 212 °C appears for all the analyzed EMC molded samples. This probably implies to the melting point of unreacted dicyandiamide [20]. The slightly exothermic increase of the heat flow between 140 °C and 210 °C indicates that the prepared EMCs were not entirely cured during the molding process. The endothermic peak of unreacted dicyandiamide may vary for different samples of EMCs, since the curing process was not terminated for any of the EMCs. The tested molded products were additional treated by a post curing thermal process to ensure the complete curing reaction of EMCs.

Figure 3c illustrates the results of DSC analysis for EMCs post cured final test products, showing well thermal stability for all prepared EMCs up to 210 °C. The thermal decomposition temperature for EMC 4 with fused silica type filler is a bit lower than others, for which the thermal degradation starts at around 219 °C, as evident from Table 2.

For our prepared EMCs, it is clear that the glass transition temperature before molding process (T_g_) remained the same and the temperature of degradation (T_decomposition_) only moderately changed for all of the tested samples. Hence, with adding different types of silica-based fillers, we were not able to significantly increase these two important parameters in the preparation of EMCs.

### 3.2. Rheology Analysis

**Figure 4:**
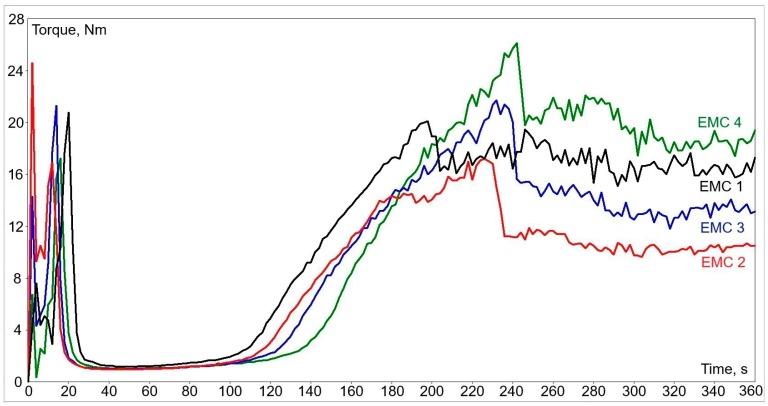
Flow-curing behaviors of prepared EMC granulates.

The resulting plastograms of all the prepared EMCs (Figure 4) illustrate the torque outline over the measuring time and show structural changes of the material. The significant values of this evaluation are the torque minimum, the residence time, and the reaction time, which are represented in Table 3 for each tested EMC granulate with the corresponding type of filler. The torque minimum describes the melt viscosity of a molded test samples before curing. The EMC 1 with glass beads type filler has for about 20% higher viscosity than other EMCs had, which is a nearly similar value. The residence time defines the duration of the melted EMC test samples before the curing process. The stable value of torque after reaching its maximum peak marks the ending of curing reaction. The time in which the process reaches that value is represented as the reaction time in which the curing process of tested EMC is finished. In our case, the residence and the reaction time were the highest for EMC 4 with the fused silica type filler and the lowest for EMC 1 with glass beads type filler.

### 3.3. Flow Spiral Length

Table 5 shows the test results. The flow spiral length of EMC with fused silica type filler is the longest, whereas the EMC with glass beads type filler is the shortest. This indicates that EMC filled with fused silica flows better in the molding process due to its smaller particles, which reduces the flow resistance. On the other hand, when considering the results of rheology analysis, increasing viscosity of EMC can reduce the flow spiral length due to the solid nature of filler type, irrespective of SiO_2_ weight content in the filler. This is also the reason that the flow spiral length of EMC 1 with glass beads type filler is evidently shorter than for others EMCs.

The bulk density of filler and the size of filler particles also affect the flow spiral length of the EMCs. The bulk densities of crystalline silica and fused silica are 1.06 g/cm^3^ and 0.60 g/cm^3^, respectively (Table 1). For the same weight, the volumetric amount of filler added to EMC of crystalline silica is lower than that of the fused silica. That is, the volumetric filling of EMC 3 prepared with crystalline silica is lower than EMC 4 prepared with fused silica. Therefore, the flow spiral length of EMC 3 with crystalline silica is shorter than EMC 4 with fused silica. Additionally, crystalline silica tends to have a larger flow resistance because of the irregular shapes with sharp edges.

### 3.4. Mechanical Properties

Table 4 lists the dimensional analysis of EMCs final products for after the molding and post curing process. From the presented data, we can notice the dimensional stability (measured with micrometer and Vernier caliper) of all tested EMCs final products very well. Table 5 summarizes the tensile strengths of all the prepared EMCs products, together with the measured densities for after molding and post curing products.

The densities of EMCs final test products very slightly decrease (around 0.5%) for all EMCs with different types of fillers after the post curing process. The density of the EMC 4 final product with fused silica filler is evidently lower than others, since this filler also has the lowest bulk density of all the used types of filler. EMC 2 final product with foundry sand filler has similarly the highest density value, as this filler has the highest bulk density among all the selected fillers. The EMC 3 final product with crystalline silica filler has for about 1.5% higher density than the EMC 1 final product with glass beads filler, despite the fact that the bulk density of filler F3 is around 20% lower than of filler F1. The specific surface area of filler F3 is still more than two times higher than of filler F1. The ability of the filler F3 to bond with the matrix binder could thus be expanded in comparison to filler F1. The density of EMC 3 final product can thereby also be higher than the EMC 1 final product because of a more tightened up filler-matrix interconnection.

The tensile strengths of EMCs final products that were prepared with glass beads, foundry sand, and crystalline silica are comparable and they are larger for about 15% than the EMC 4 final product that was prepared with fused silica. The EMC 2 final product does not exhibit the best mechanical properties, but it is composed of the cheapest and more accessible of all the selected silica-based fillers. The larger tensile strength of the EMC 3 final product might as well be attributed to good filler-matrix interaction. The ability of the filler to bond with the matrix was likely to be enhanced due to a more defined shape of filler. Another possible reason for the reduction of the tensile strength for EMC 4 final product with fused silica filler is its higher specific surface area due to its small particle size and tendency to agglomerate, which results in lower surface bonding between the matrix and the filler.

## 4. Conclusions

The effects of filler types containing silicon dioxide on the properties of epoxy molding compounds are experimentally investigated in this study for four different fillers. The key findings are as follows:

(1) Irrespective of filler types, T_g_ and curing temperature remain almost the same for all EMCs granulates, whereas the EMC final product with glass beads filler has the highest thermal decomposition temperature of all the presented EMCs.
(2) The viscosity of EMCs molded granulates is the highest for glass beads type filler, while the others have comparable viscosity.
(3) The flow spiral length of EMCs with different fillers in descending order is fused silica > foundry sand > crystalline silica > glass beads.
(4) The tensile strength of EMCs final products with glass beads, foundry sand, and crystalline silica is nearly the same and it is independent of SiO_2_ weight content in the filler. The tensile strength for these EMCs final products is higher than for the EMC with fused silica.

An overall comparison reveals that various types of fillers with different SiO_2_ content and different particle size can influence on the flowability of EMCs, hence, with smaller particle size, we can gain better flowability. The obtained results on the glass beads, foundry sand, and crystalline silica type fillers show better mechanical properties of the final composite product than the fused silica. The tensile strength for EMC with fused silica filler is clearly about 15% lower as others. EMC with glass beads type filler has quite high tensile strength value and it is the most appropriate composite for industrial utilization. Due to its shortest reaction time, glass beads type of silica filler is able to contribute to a reduction of production costs.

As a result, we would be able to ensure that the industrially applied EMC final product with our selection of silica-based filler is going to achieve the same qualities as the EMC final product that was prepared in our research. Additional tests on the injection, transfer, or compression molding operation are required to complete the optimal scale-up of a pilot line and to promote further scale-up processing for the industrial design of the EMC production.

## Figures and Tables

**Table 1:** Weight content of silicon dioxide, bulk density and specific surface area of the tested fillers.

Filler Mark	Type of Filler	Weight Content of SiO_2_, %	Bulk Density, g/cm^3^	Specific Surface Area, m^2^/g
F1	Glass beads	68.00–75.00	1.36	0.38
F2	Foundry sand	15.32–21.60	1.83	0.11
F3	Crystalline Silica	≈99.70	1.06	0.86
F4	Fused Silica	≈99.70	0.60	4.94

**Table 2:** Temperature results of DSC thermogrames for prepared EMCs.

Epoxy Molding Compound	Filler Mark	T_g_, °C	T_curing peak_, °C	T_decomposition_, °C
EMC 1	F1	113.8	170.4	221.0
EMC 2	F2	113.0	174.1	218.9
EMC 3	F3	113.8	174.1	217.8
EMC 4	F4	114.7	171.6	214.0

**Table 3:** Evaluations of torque rheometer plastograms for each EMC granulate.

Epoxy Molding Compound	Filler Mark	Torque Minimum, Nm	Residence Time, s	Reaction Time, s
EMC 1	F1	1.2	87	154
EMC 2	F2	0.9	99	162
EMC 3	F3	1.0	109	191
EMC 4	F4	1.0	122	202

**Table 4:** Dimensional measurement of EMCs final test products for after molding and post curing process.

		After Molding Process			Post Curing Process		
Epoxy Molding Compound	Filler Mark	Outer Diameter, cm	Inner Diameter, cm	Height, cm	Outer Diameter, cm	Inner Diameter, cm	Height, cm
EMC 1	F1	19.93	12.033	14.99	19.93	12.036	14.99
EMC 2	F2	19.92	12.050	14.98	19.92	12.045	14.99
EMC 3	F3	19.92	12.044	14.97	19.92	12.046	14.98
EMC 4	F4	19.92	12.038	14.98	19.93	12.042	14.98

**Table 5:** Physical and mechanical properties of tested EMCs granulates and final test products.

Epoxy Molding Compound	Filler Mark	EMMI, inch	Density AM, g/cm^3^	Density PC, g/cm^3^	Tensile Strength, N
EMC 1	F1	19.6 ± 1.2	1.904	1.894	1910 ± 130
EMC 2	F2	23.9 ± 0.4	1.967	1.958	1850 ± 120
EMC 3	F3	21.1 ± 0.6	1.934	1.924	1990 ± 240
EMC 4	F4	24.4 ± 0.7	1.876	1.864	1680 ± 200
